# Application of digital and intelligent intervention in medication errors of metformin after CTA examination

**DOI:** 10.3389/fmed.2026.1874553

**Published:** 2026-08-17

**Authors:** Ting Zhao, Yan Xu, Haihong Li, Qiuyu Gu, Min Shi, Yu Liu, Meng Wu

**Affiliations:** The First People’s Hospital of Lianyungang, Lianyungang, China

**Keywords:** CTA examination, digital and intelligent, intervention, medication error, root cause analysis

## Abstract

**Objective:**

To explore the effectiveness of digital and intelligent intervention in the medication errors of metformin after CTA examination.

**Methods:**

Patients who underwent CTA examinations in a tertiary A general hospital in Jiangsu Province from January to September 2025 were selected as the study subjects. The patients from January to April 2025 who received intervention based on conventional information technology were in a control group, and the patients from June to September 2025 who received digital and intelligent intervention were in an intervention group. The incidence of metformin misadministration, the satisfaction of medical staff, the pass rate of the assessment on medication contraindications for CTA examinations, the participation rate of caregivers, the compliance rate of nurses in distributing oral medications, and the number of oral medication errors were compared between the 2 groups.

**Results:**

After applying digital and intelligent intervention, the incidence of metformin overdose in CTA examination patients decreased from 2.36 to 0.32%. There was a statistically significant difference between the intervention group and the control group (χ^2^ = 85.340, *P* < 0.001). The satisfaction rate of medical staff was 98.41%, which was higher than 82.22% before the intervention (χ^2^ = 132.405, *P* < 0.001). The pass rate of medical staff’s assessment on CTA examination medication contraindications was 96.60%, which was significantly higher than 76.16% before the intervention (χ^2^ = 126.103, *P* < 0.001). The participation rate of CTA examination patients’ caregivers increased from 46.71 to 96.67% (χ^2^ = 3390.581, *P* < 0.001). The compliance rate of nurses in distributing oral medications increased from 90.03 to 98.53% (χ^2^ = 40.972, *P* < 0.001). The number of oral medication errors decreased from 3 cases before the intervention to 1 case after the intervention.

**Conclusion:**

Digital and intelligent intervention can reduce medication errors of metformin after CTA examination for patients. These interventions help decrease the incidence of adverse events, promote the involvement of both patients and caregivers, optimize the healthcare service system, and ensure patient safety.

## 1 Introduction

Computed Tomography Angiography (CTA) is an important method for screening and diagnosing cardiovascular diseases, and its clinical application is increasingly widespread (1). The use of iodine-containing contrast agents is a necessary step in completing CTA examinations, but their impact on renal function cannot be ignored. Research (2) indicates that iodine-containing contrast agents may cause contrast-induced nephropathy, resulting in transient or persistent renal function decline. Metformin, as the first-line treatment drug for type 2 diabetes, is widely used in clinical practice (3). When renal function is impaired, the metabolism of metformin is blocked, and it is prone to accumulate in the body, increasing the risk of fatal lactic acidosis (4). Therefore, multiple studies (5, 6) at home and abroad suggest that patients with abnormal renal function should suspend the use of metformin 48 h before and after CTA examination to avoid the combined risk of kidney damage and drug accumulation.

Although there are clear medication contraindications, in the busy clinical work, the mode relying on the manual memory and process execution of medical staff has significant loopholes. If there are any omissions in the connection of multiple links such as doctor’s orders, drug suspension, patient education, and drug distribution, it is very likely that patients will accidentally take metformin within 48 h after CTA examination, posing a serious safety hazard. The traditional management model mainly focuses on post-case handling and personnel education, which is difficult to fundamentally solve systematic problems (7). The integrated root cause analysis method (RCA) as a systematic problem tracing tool can penetrate the surface reasons and directly reach the defects in the process and system design, providing scientific basis for continuous quality improvement (8). Since 2014, our hospital has carried out nursing informatization, closely following the pace of informatization construction, continuously integrating information technology with medical work, and establishing a smart hospital information system. However, there are still problems such as low data utilization and insufficient decision support. In this study, the deep integration of information technology and intelligent tools provides the possibility for the construction of an “active defense system combining human defense and technical defense.” Based on the system logic of “if… then…,” multiple systems are linked together to form an automated rule engine of “identification—decision-making - execution,” whose core is the rule reasoning engine of the clinical decision support system. It continuously monitors the prescribing and execution behaviors of doctors and nurses. When a doctor issues a CTA examination prescription for a patient in the system, it becomes a triggering signal. The engine immediately “identifies” this instruction, and automatically extracts the current medication record and recent renal function test results of the patient from the electronic medical record, and initiates the risk assessment process. When a nurse uses a PDA to execute the prescription, the system will perform mandatory verification. If the prescription has been marked as “not to be executed,” the scanning process will require a second confirmation; if metformin is attempted to be dispensed within 48 h after the CTA examination, the system will directly intercept and prompt the risk, and if it is necessary to continue the medication, a reason must be filled in for filing. All warnings and operation data are captured in real time by the background, and the rectification effect is intuitively displayed through the data analysis dashboard, and high-risk links or personnel are identified. Digitalization is achieved through the integration of artificial intelligence, mobile medical, etc., driven by digitalization and intelligence, and has the characteristics of intelligence, real-time, and quantifiability (9).

This study uses digitalization intervention to reduce metformin medication errors after CTA examination, continuously promoting the transformation of information technology support from “process support” to “assisted decision-making,” in order to provide a feasible practical solution for reducing similar medication errors and ensuring patient safety.

## 2 Materials and methods

### 2.1 Study design and participants

The patients who underwent CTA examinations in the tertiary grade A general hospitals of Jiangsu Province from January to September 2025 was selected as the study subjects. The 6,224 patients who underwent CTA examinations based on the conventional information system in January to April 2025 were taken as the control group, and the 5,316 patients who underwent CTA examinations using the digital intelligence intervention method from June to September 2025 were taken as the intervention group.

Inclusion criteria included: (1) Patients who underwent CTA examinations as per the doctor’s advice; (2) age > 18 years. Exclusion criteria: (1) patients with urgent or critical conditions; (2) patients with existing or previous mental disorders. This study did not involve the privacy and privacy data of the research subjects. After discussion by the hospital’s ethics committee, it was decided to waive the ethical review.

This retrospective before-after comparative study included all patients satisfying the inclusion and exclusion criteria during the predefined pre-intervention and post-intervention periods.

### 2.2 Research methods

The control group patients underwent the CTA examination process utilizing a conventional information-based method. For patients who inadvertently took metformin during the same period as the CTA examination, a retrospective analysis was conducted using an integrated RCA. In contrast, patients in the intervention group completed the CTA examination process through a digitalized intervention method.

#### 2.2.1 Event determination

On April 10, 2025, a physician in a specific ward ordered a CTA examination for a patient. The on-duty nurse verified this order and subsequently issued the CTA examination form to both the patient and the caregiver, providing relevant educational information. Concurrently, the nurse noticed that the oral medication order for metformin had not been canceled. She promptly reminded the physician to discontinue the long-term oral metformin prescription. However, after the physician canceled the order, the nurse did not immediately remove the discontinued metformin from the oral medication bag. During the night shift, the nurse checked the medications against the oral medication packages and the personal digital assistant (PDA) used for medication administration. Utilizing the PDA, she distributed the oral medications. Prior to the night shift’s medication administration, the caregiver discovered metformin among the distributed oral medications and consulted the on-duty nurse manager. The nurse manager provided an explanation and reassurance to both the patient and the caregiver, and initiated an investigation into the incident. Upon learning of the occurrence, she promptly posted a message in the work group to alert everyone and reiterated the importance of the matter during the morning meeting. From January to April 2025, a total of 6,224 patients in the hospital underwent CTA examinations as ordered by the physicians. Among these patients, 147 mistakenly ingested metformin within 48 h following the CTA examination, with cases distributed across 13 wards, including trauma surgery and hepatobiliary surgery. Utilizing the abnormal event decision tree, this sequence of events was identified as a systemic issue. Based on the severity of the incident, the frequency of recurrence, and the criteria outlined in the risk matrix assessment, the incident was classified as level 3. A root cause analysis was conducted immediately.

#### 2.2.2 Incident investigation

##### 2.2.2.1 Establishment of the RCA team

The special team consists of two parts: the expert team and the event-related team, with a total of 14 members. The members of the expert team include the head of the nursing department, the head of the quality control department, the director of the Quality and Safety Management Office and his deputy, the deputy director of the Medical Department, the head of the Information Department, the director of the Imaging Department, the deputy director of the Pharmacy Department, and the head of the departmental nurses. The event-related team consists of the department head, head nurse, and 3 specialized nurses. The expert team is the main responsible group, possessing RCA expertise. All members of the team have received comprehensive RCA training and are familiar with the work process. They can conduct independent investigations and have critical analysis capabilities.

##### 2.2.2.2 Event review and data collection

The special team was composed of an interview group (5 people), a process review group (4 people), and a document review group (3 people). After the incident occurred, the interview group conducted interviews with on-site witnesses who had a direct connection to the event; the process review group conducted on-site inspections to check the medical equipment used and the environment of the wards; the document review group comprehensively recorded the relevant documents based on the characteristics of the case and the analysis requirements, and generated a chronological list.

##### 2.2.2.3 Identify the proximate cause

Members of the RCA team identified the proximate causes from five aspects: personnel, equipment, environment, materials, and systems by using a fishbone diagram. The details are shown in Table 1.

**TABLE 1:** The proximate cause of the patient’s incident of accidental ingestion of metformin within 48 h during the CTA examination.

Number	Proximate cause
1	Doctors and nurses were not aware that the oral metformin medication needed to be discontinued 48 hours after the CTA examination.
2	After the CTA examination, if it was not within 48 hours, the doctor prescribed metformin.
3	The imaging department staff informed the patient and their caregivers to stop taking the medication, but the patient and their caregivers did not take it seriously.
4	The doctor did not check the kidney function results or whether the patient was taking metformin before ordering the CTA examination.
5	The nurse did not explain the precautions for the special examination when distributing the checklist.
6	After the caregiver relayed the information to the doctor, the doctor did not suspend the treatment plan/ did not inform the nurses or the radiology department.
7	The head nurse emphasized the risks but failed to monitor whether the doctor’s orders had been stopped.
8	The nurses in the office department failed to promptly verify the updated long-term medical orders and crossed out the drugs that were discontinued from the oral medication list.
9	The doctor stopped the medication later than the completion of the examination, with the delay exceeding 20 hours.
10	The bedside nurse failed to properly hand over the special medical instructions to the night shift nurse.
11	When distributing oral medications, the specific drugs were not checked, resulting in missed inspections.

##### 2.2.2.4 Identification of root cause

**FIGURE 1:**
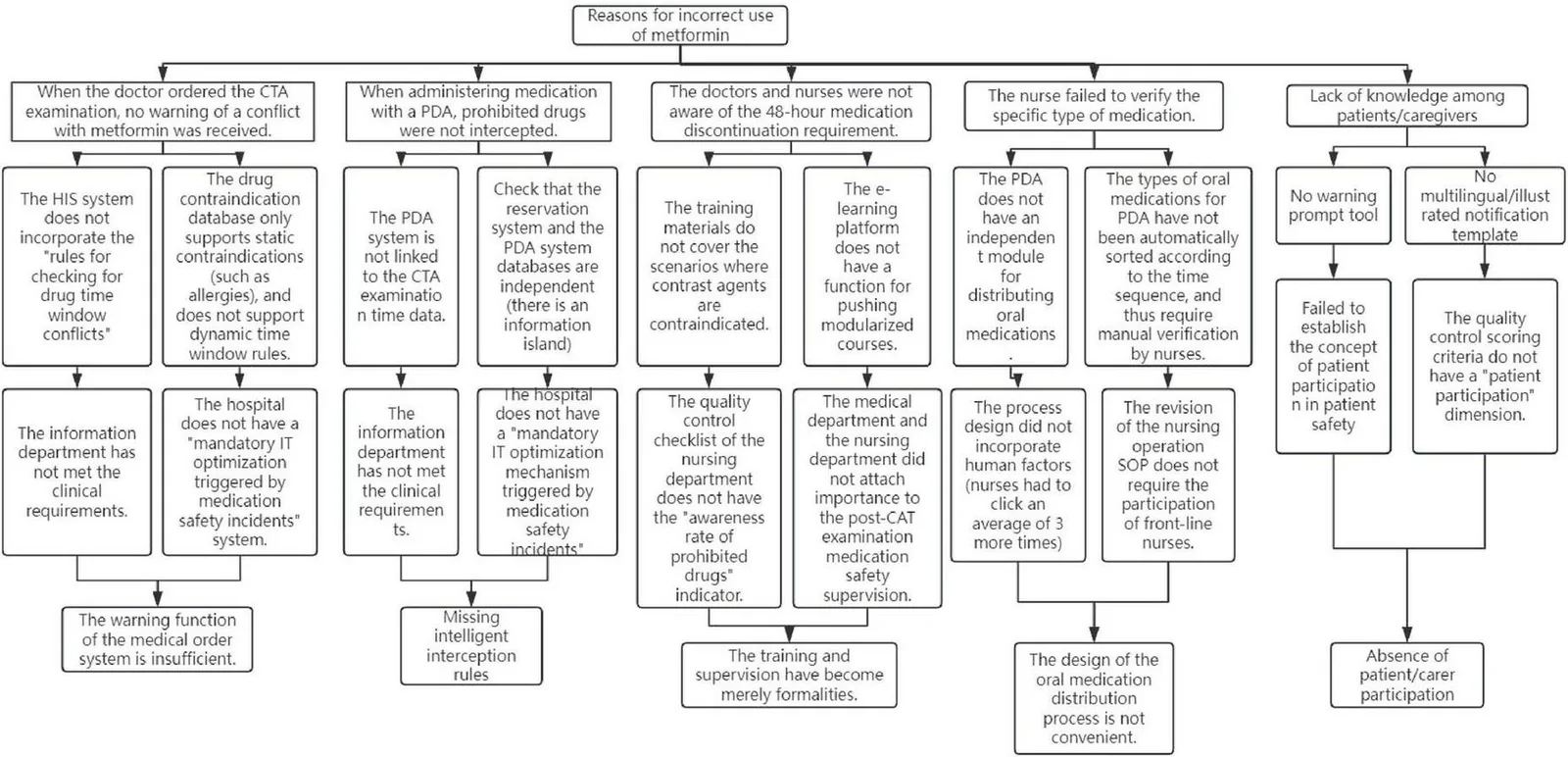
“5WHY” CTA was used to conduct a root cause analysis of the incident where a patient accidentally ingested metformin within 48 h.

The “5WHY” method is used to further identify the root cause. The following questions are used for determination: If this cause no longer exists, is the problem likely to still occur? If this cause is corrected, will the problem still occur? If this cause is eliminated, will similar incidents recur? If the answers to all are no, then it is the root cause. After analysis, the root cause is finally confirmed (see Figure 1 for details).

### 2.3 Intervention plan

#### 2.3.1 The control group adopts the conventional informationization method

After the physician issued the CTA medical order, the nurse utilized the PDA check module to distribute the examination form and provided instructions regarding the relevant precautions associated with the form to both the patient and the caregiver. Additionally, she advised the patient to discontinue the use of metformin 48 h prior to and following the CTA examination. In cases where the patient has been on this medication for an extended period, she also notified the physician to discontinue the metformin prescription.

#### 2.3.2 The intervention group implemented a digital and intelligent intervention plan based on RCA

**FIGURE 2:**
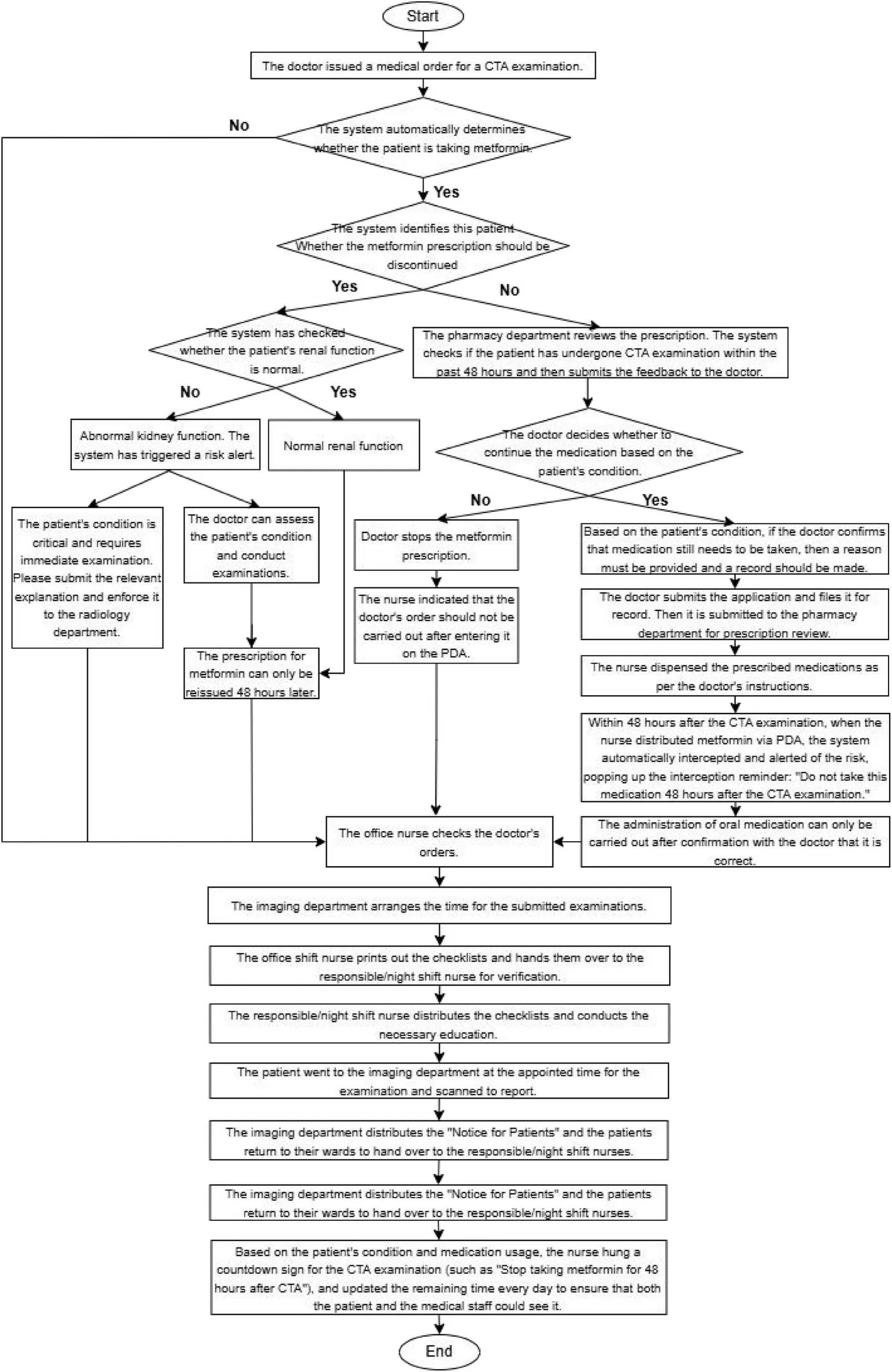
Digital and Intelligent Intervention Flowchart.

The digital and intelligent intervention flowchart is shown in Figure 2.

##### 2.3.2.1 A human-machine collaborative “strong early warning” system was established to mitigate 90% of human error risks

(1) Pre-event reminder: Through the Clinical Decision Support System (CDSS), pop-up prompts are integrated into the physician’s workflow. When a physician issues a CTA prescription, the system automatically verifies whether the patient is currently prescribed metformin and subsequently displays a risk alert: “It has been detected that this patient has a prescription for oral metformin. Please monitor the patient’s renal function. If abnormalities are present, the patient should discontinue metformin for 48 h prior to undergoing the CTA examination. For patients with normal renal function who are taking metformin, it is advised to cease the medication 48 h after the CTA examination.”

(2) In-process interception: ➀ Before improvement: On the PDA execution end, when a doctor long-presses an unexecuted prescription, a prompt “Reason for not executing the prescription” will pop up. Select or enter the reason based on the actual situation. This prescription will change from the red “pending execution” status to the gray “not executed” status. If the doctor continues to scan this prescription, it will directly change to the green “executed” status. After improvement, optimize the “gray” prescription function: When a nurse points to a gray prescription and indicates not to execute, if the doctor continues to scan this prescription, a prompt “Please cancel the not-executed prescription status first!” will pop up. The execution of the prescription needs to be confirmed by the nurse twice before continuing to scan. ➁ To avoid situations where CTA examination prescriptions cannot be issued in special circumstances, no restrictions are imposed. Instead, warning reminders are provided. Establish intelligent interception rules: Within 48 h after the CTA examination, when the nurse distributes metformin via PDA, the system automatically intercepts and alerts risks, displaying an interception reminder: “48 h after the CTA examination, metformin cannot be taken.” If, based on the patient’s condition, the doctor still needs to continue taking the medication, reasons need to be filled in and a record made.

(3) Post-event traceability: Based on the hospital’s big data center, develop an operational decision analysis system module to capture and display the rectification effects in real time.

##### 2.3.2.2 Establish a three-level physical defense line of “radiology department—ward—caregiver” to block the process breakpoints

(1) Front-end double-signature confirmation: Design the “Informed Consent Form for Enhanced CT Examination,” including the examination time, medication cessation requirements, and risk explanations, with both the patient and the volunteer signing.

(2) Mid-level visual warning: Produce a bedside-mounted CTA examination countdown reminder sign (such as “48 h after CTA, suspend taking metformin”), and have the nurse update the remaining time daily to ensure that both the patient and the medical staff can see it.

(3) Back-end caregiver coordination: After the examination, the imaging department will distribute the instructions card to the patients, who will then take it back to their wards and hand it over to the bedside nurse.

##### 2.3.2.3 “Dual dimension of training supervision and anti-defect design” to reduce the risk of medication errors

**FIGURE 3:**
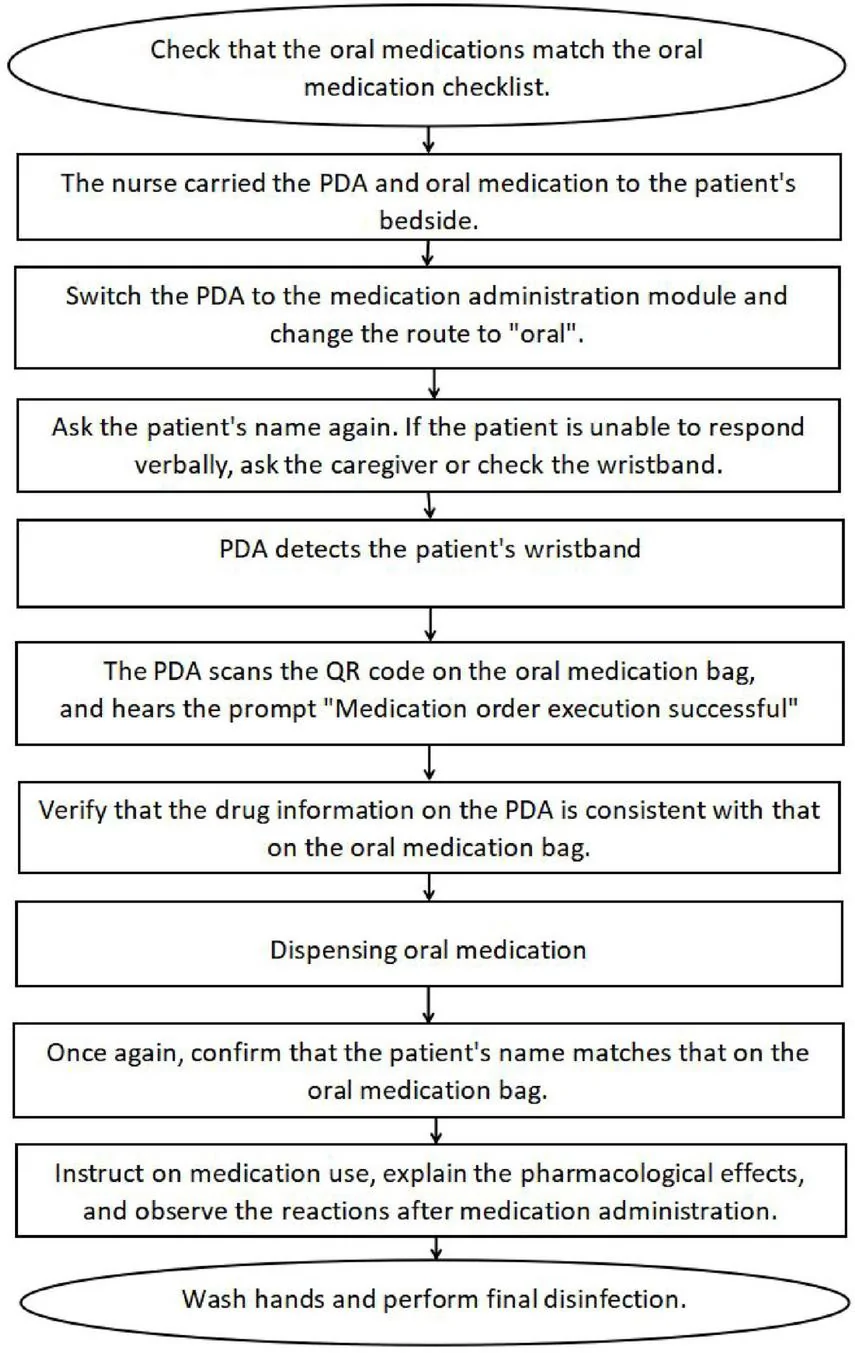
The oral medication dispensing process of PDA before optimization.

**FIGURE 4:**
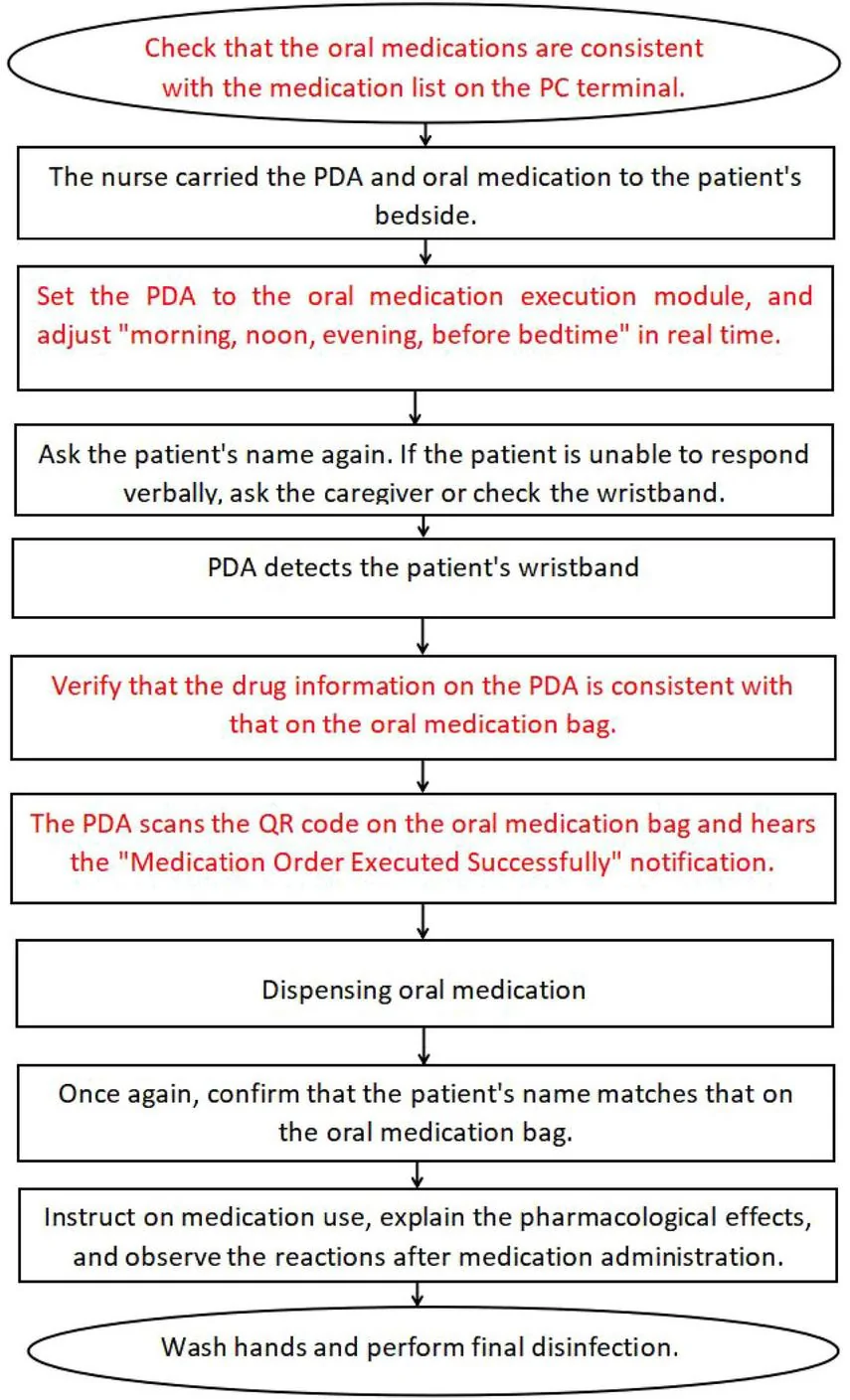
Oral medication dispensing process for optimized PDA.

(1) Develop an “CTA examination post-medication contraindications” practical training course, including simulation scenarios (such as educating CTA examination patients about the necessity of suspending metformin), and nurses need to complete the entire process from “identifying risks” to “executing discontinuation” in the simulation system.

(2) Adopt the “four no’s and two direct” (no notification, no greeting, no report, no accompaniment, directly to the department, directly to the scene) approach for key inspections.

(3) Optimize the PDA module (barrier-free design): Before improvement, the PDA process of issuing oral medications had the following problems: ➀ The paper-based oral medication list required manual synchronization with the doctor’s orders, which might be delayed due to the office staff’s handling of the doctor’s orders, resulting in insufficient execution of the doctor’s orders; ➁ In the PDA module, the issuance of oral medications was included in the medication administration process, and it was necessary to switch the process to select the oral medication issuance during the medication administration process, and it was impossible to clearly see all the drugs at special time points such as morning, noon, and evening; ➂ The linear operation design of scanning first and then verifying exists a cognitive load risk; ➃ It was not linked to the time axis of the doctor’s order system and relied on the nurse’s personal experience judgment, which was prone to omission due to negligence. In response to the above problems, combined with human factors engineering principles, the system was improved from “usable” to “willing to use,” and the PDA process of issuing oral medications was refined (see Figures 3, 4): ➀ Cancel the paper list: When nurses check the oral medications, they must use the PC end to real-time retrieve the changes in the doctor’s orders to ensure synchronization with the latest doctor’s orders; ➁ From “usable” to “easy to use”: A separate module design was created, a new independent “oral medication issuance” module was established, and the selection of special time points was added to facilitate use and be clear at a glance; ➂ From “easy to use” to “good to use”: The system filtered out the drugs that were not in the issuance period based on the current time; if it was ahead of or behind the issuance time, a pop-up window would warn “Not yet/Passed the issuance time”; ➃ From “good to use” to “willing to use”: The background set up a PDA security guard to monitor the oral medication issuance data in real time, screen out high-risk nurses, and the information platform could retrieve various doctor’s order scanning execution situations.

### 2.4 Evaluation indicators

Four specialist nurses from the RCA team conducted the investigation and analysis of various outcome indicators. ➀ The incidence of metformin misadministration within 48 h after CTA examination was calculated as the number of patients with metformin medication errors within 48 h after CTA examination/total number of CTA examination patients × 100%. ➁ Satisfaction was evaluated using a self-developed satisfaction questionnaire, including 3 items: system usage standardization, operational convenience, and system layout rationality. The number of “unsatisfied,” “basically satisfied,” and “satisfied” was counted respectively, and the number of staff in the department where metformin medication errors occurred after CTA examination who were satisfied was calculated/total number of department staff × 100%. ➂ The pass rate of medical staff’s knowledge assessment on CTA examination medication contraindications, using a self-developed CTA examination medication contraindications test paper, for all medical staff in the department where metformin medication errors occurred after CTA examination. ➃ The participation rate of caregivers of CTA examination patients. ➄ The standardization rate of nurse-administered medication, through on-site inspection using a self-made standard for nurse-administered medication standardization, and tracking the data on oral medication administration through the information platform, statistics were made on the standardized situation of nurse-administered medication before and after the intervention, and the calculation method was the number of standardized cases of nurse-administered medication/total number of nurse-administered medication cases. ➅ The number of oral medication errors.

### 2.5 Statistical analysis

The data were analyzed using SPSS 26.0 statistical software. Count data were described by frequency and percentage, and the comparison between groups was conducted using the χ^2^ test. A difference was considered statistically significant when *P* < 0.05. Multivariable logistic regression was used to assess the association between the digital intervention and metformin-related medication errors, adjusting for age, sex, baseline eGFR category ( ≥ 60, 45–59, 30–44, and < 30 mL/min/1.73 m^2^), CTA scheduling status (elective vs. emergency), and ordering shift (day vs. night). Adjusted odds ratios (ORs) with 95% confidence intervals (CIs) were reported. Firth penalized logistic regression was additionally performed as a sensitivity analysis to reduce potential sparse-data bias (10).

## 3 Results

### 3.1 Comparison of the incidence of metformin administration errors within 48 h after CTA examination between the two groups of patients

The intervention group consisted of 5,316 cases, with 17 cases of metformin medication errors, an incidence rate of 0.32%; the control group had 6,224 cases, with 147 cases, an incidence rate of 2.36%; compared between the intervention group and the control group, the difference was statistically significant (χ^2^ = 85.340, *P* < 0.001), as shown in Table 2.

**TABLE 2:** Comparison of the occurrence of metformin mis-administration within 48 h after CTA examination between the two groups [*n* (%)].

Group	Occurrence	Not occurrence	χ^2^-value	*P*-value
Control group	147(2.36)	6077(97.64)	85.340	<0.001
Intervention group	17(0.32)	5316(99.68)		

### 3.2 Comparison of medical staff satisfaction before and after digital and intelligent intervention

A survey was conducted among the medical staff in the departments where metformin medication errors occurred after CTA examination. The results showed that a total of 883 medical staff were surveyed. The number of staff who were satisfied increased from 726 before the intervention to 869 after the intervention. In summary, the results showed that after digitalization intervention, the satisfaction rate of medical staff was 98.41%, which was higher than 82.22% before the intervention (χ^2^ = 132.405, *P* < 0.001), as shown in Table 3.

**TABLE 3:** Comparison of medical staff satisfaction before and after digital and intelligent intervention [*n* (%)].

Group	Satisfied	Not satisfied	χ^2^-value	*P*-value
Control group	726(82.22)	157(17.78)	132.405	<0.001
Intervention group	869(98.41)	14(1.59)		

### 3.3 Comparison of the pass rate of the knowledge assessment on medication contraindications for CTA examinations among medical staff before and after digital and intelligent intervention

In total, 883 medical staff were evaluated. Before the intervention, 699 of them passed the assessment on the knowledge of medication contraindications for CTA examinations. After the intervention, 853 of them passed the assessment. The qualified rate of medical staff’s knowledge assessment on contraindications for CTA examination was 96.60%, which was significantly higher than the previous 79.16% (χ^2^ = 126.103, *P* < 0.001), as shown in Table 4.

**TABLE 4:** Comparison of knowledge assessment on contraindications for CTA examination medication among medical staff before and after digital and intelligent intervention [*n* (%)].

Group	Pass	Failed	χ^2^ *-*value	*P*-value
Control group	699(79.16)	184(20.84)	126.103	<0.001
Intervention group	853(96.60)	30(3.40)		

### 3.4 Comparison of caregiver participation before and after digital and intelligent intervention

The survey was conducted to investigate whether the caregivers of 5,316 patients in the intervention group and the caregivers of 6,224 patients in the control group participated in the patients’ examinations. The results showed that the participation rate of caregivers increased from 46.71% before the intervention to 96.67% after the intervention (χ^2^ = 3390.581, *P* < 0.001) (see Table 5 for details).

**TABLE 5:** Comparison of caregiver participation before and after digital and intelligent intervention [*n* (%)].

Group	Participated	Not participated	χ^2^-value	*P*-value
Control group	2907(46.71)	3317(53.29)	3390.581	<0.001
Intervention group	5139(96.67)	177(3.33)		

### 3.5 Comparison of oral medication distribution norms of nurses before and after digital and intelligent intervention

This study investigated the oral medication distribution norms of 612 nurses. The results showed that the number of nurses adhering to the oral medication administration norms increased from 551 before the intervention to 603 after the intervention. The rate of oral medication distribution norms by nurses was 98.53%, which was higher than 90.03% before the intervention (χ^2^ = 40.972, *P* < 0.001). The details are presented in Table 6.

**TABLE 6:** Comparison of oral medication distribution norms of nurses before and after digital and intelligent intervention [*n* (%)].

Group	Normative	Non-normative	χ^2^-value	*P*-value
Control group	551(90.03)	61(9.97)	40.972	<0.001
Intervention group	603(98.53)	9(1.47)		

### 3.6 The number of cases where oral medication was administered incorrectly

By comparing the number of oral medication administration errors before and after the intervention, the results showed that the number of oral medication administration errors decreased from 3 cases before the intervention to 1 case after the intervention, indicating a significant reduction.

### 3.7 Results of multi-factor analysis

After adjustment for potential covariates, the digital intervention remained significantly associated with lower odds of medication error (adjusted OR, 0.123; 95% CI, 0.067–0.227; *P* < 0.001) (see Table 7 for details). The Firth penalized logistic regression sensitivity analysis yielded a consistent estimate (adjusted OR, 0.128; 95% CI, 0.067–0.225; *P* < 0.001), as shown in Table 8.

**TABLE 7:** Multivariable logistic regression analysis of factors associated with metformin-related medication errors.

Predictor	Adjusted OR	95% CI lower	95% CI upper	*P*-value
Digital intervention (vs. conventional)	0.123	0.067	0.227	< 0.001
Age (per 10-year increase)	0.950	0.792	1.139	0.577
Female (vs. male)	0.848	0.513	1.402	0.521
eGFR 45–59 (vs. ≥ 60)	0.306	0.042	2.232	0.243
eGFR 30–44 (vs. ≥ 60)	0.548	0.075	4.025	0.555
eGFR < 30 (vs. ≥ 60)	1.572	0.619	3.993	0.342
Emergency (vs. elective)	0.959	0.519	1.772	0.894
Night shift (vs. day shift)	0.969	0.457	2.055	0.934

**TABLE 8:** Firth penalized logistic regression sensitivity analysis of factors associated with metformin-related medication errors.

Predictor	Adjusted OR	95% CI lower	95% CI upper	*P*-value
Digital intervention (vs. conventional)	0.128	0.067	0.225	< 0.001
Age (per 10-year increase)	0.945	0.793	1.140	0.546
Female (vs. male)	0.856	0.514	1.397	0.538
eGFR 45–59 (vs. ≥ 60)	0.454	0.051	1.695	0.282
eGFR 30–44 (vs. ≥ 60)	0.812	0.091	3.062	0.797
eGFR < 30 (vs. ≥ 60)	1.703	0.618	3.815	0.275
Emergency (vs. elective)	0.986	0.518	1.749	0.963
Night shift (vs. day shift)	1.016	0.455	2.002	0.965

## 4 Discussion

### 4.1 The implementation of digital and intelligent intervention measures is correlated with a decrease in adverse event rates, enhanced control of potential risks, and improved patient safety levels

The observed findings indicate that the implementation of digital and intelligent intervention is associated with a reduction in metformin-related medication errors within 48 h following CTA scans and a decline in associated latent risks. Meanwhile, the digital and intelligent intervention is also correlated with improved awareness of relevant protocols among clinical staff and a higher engagement level of patients’ caregivers during CTA examinations. Importantly, this igital and intelligent intervention is not the sole determinant driving these improvements; instead, it serves as a key facilitator functioning synergistically within the broader socio-technical healthcare ecosystem of the hospital. This study directly addresses the key issues in the proximal causes, such as “the absence of mandatory interception function in the information system” and “the reliance on manual verification at the nurse’s end,” by constructing a full-chain human-machine collaborative early warning system; the pop-up prompts of CDSS at the doctor’s end place the knowledge of medication contraindications at the decision-making starting point; the optimization of the “point gray” doctor’s orders function on the PDA and the intelligent interception during distribution, through information technology means to set hard checkpoints, maximize the elimination of human errors caused by work overload, memory deviation or operational negligence, which is highly consistent with the anti-guessing principle advocated in modern safety management, “making errors difficult to occur through design” (11). The post-tracing module is established to achieve continuous monitoring of quality and data-driven management. Additionally, the “imaging department—ward—caregiver” three-level physical defense line constructed by this study, through double-sign informed consent forms, bedside visual reminder signs and paper notification cards, etc., multiple senses and multiple scenarios of repeated reminders, strengthens the transmission and confirmation of key information among patients, caregivers and different department medical staff, breaks information islands, effectively blocking the breakpoints in the circulation of information between departments (12), and improves the participation and compliance of patients and caregivers. This study translates digital and intelligent intervention into specific and implementable information technology solutions, and this practice is associated with decreased adverse event incidence. It is observed that the transformation of information support from “process support” to “data-driven” mode, the realization of workflow closed-loop management (13), and ongoing targeted optimization are correlated with reduced potential risks and enhanced patient safety. This set of measures cannot be regarded as the only determinant for risk control and patient safety protection, but serves as an important collaborative component in the hospital’s comprehensive socio-technical management system.

### 4.2 The application of digital and intelligent intervention measures is associated with more standardized work practices among healthcare professionals and improved job satisfaction

The results show that the digital and intelligent intervention is linked to standardized work processes and increased satisfaction among medical staff. Digital intervention, as a crucial supporting element, plays a significant role in the hospital technical environment. Study (14) indicates that optimizing the system design can achieve precise matching of technology with the physical and mental as well as cognitive capabilities of people, thereby improving system efficiency, safety, and user experience. This study simplifies the process through anti-defect design, reduces cognitive load (such as optimizing the PDA oral medication distribution module), and simultaneously develops targeted simulation training courses, supplemented by the “four no’s and two direct” supervision method. This not only consolidates the knowledge and skills of medical staff but also reshapes their safety behavior habits. This “system optimization to reduce burden, training supervision to strengthen awareness” dual-track model helps to form a long-term safety culture. This strategy integrates human factors engineering principles and embodies the optimization idea of “human-machine combination,” which is crucial for improving medical quality and patient safety (15). Relevant research (16) shows that applying human factors engineering theory to construct a nursing human resource prediction model has strong practicality. Therefore, in the future, it is recommended to base on human factors engineering principles, continuously optimize information technology support, precisely match information technology with nursing work, continuously improve nursing efficiency, optimize resource allocation, improve patient experience, and promote the continuous transformation of nursing models toward precision, individualization, and digitalization.

### 4.3 By tracing the causes based on RCA, a digital and intelligent intervention can be constructed, which can enhance multi-departmental collaboration and coordination, and promote the refined management supported by information

This study focused on the high-risk aspects of metformin administration errors within 48 h after CTA examination. It utilized integrated RCA to trace the causes, strengthened multi-department collaboration and coordination, tracked the deficiencies in personnel, systems, and management throughout the original process, and relied on information technology to formulate multi-dimensional interventions covering process reengineering, physical warnings, and personnel training, aiming to promote information-based digitalized management. This study deeply integrated quality management tools with information technology innovations, achieving closed-loop management from problem analysis to solutions. The intervention combined technical “hard constraints” and management “soft intervention,” constructed a three-dimensional prevention network, and also paid attention to the role of caregivers in the safety defense line, expanding the participation boundaries of patient safety management. With the development of information technology, artificial intelligence has been integrated into medical services, and various intelligent devices provide full-chain support. This has improved medication safety (17). In the future, it is recommended to continue strengthening multi-department collaboration, continuously optimize medical service processes, precisely build information platforms to assist clinical work, improve medical service efficiency, and enhance the accuracy of the visit process; at the same time, continuously explore deeper integration paths of information technology and medical services, strengthen the penetration of digitalized support in all stages of diagnosis and treatment, and lay the foundation for the construction of a precise medical service system in the future.

### 4.4 Discussion on alarm fatigue based on digital and intelligent intervention

Alarm fatigue may reduce the response of frontline healthcare workers to clinical alarms (18), interfere with work efficiency, pose safety risks to patients, lead to medical errors, and in severe cases, even cause patient deaths. Therefore, it is necessary to take measures to address this critical issue (19). Research (20) shows that adopting an intelligent management approach can effectively reduce the degree of alarm fatigue, and the application of new technologies can also effectively improve the recognition of alarms, shorten the response time to alarms, reduce the frequency of false alarms, and lower the degree of alarm fatigue, thereby ensuring patient safety (21). Different from the traditional uniform and all-day alert window reminder mode, this study adopts a design scheme that combines clinical scenario screening with a hierarchical risk triggering mechanism. This system will not push reminders for events that do not meet the alarm logic, such as medication orders without an impact on CTA examinations or abnormal indicators. However, when the CTA examination order - metformin medication order - renal function status match the alarm logic and show abnormalities, the alarm work will automatically trigger targeted alarm prompts based on the situation, thereby fundamentally reducing ineffective and unnecessary alarm generation from the source. We have optimized the alarm interface and presentation method to balance the efficiency of early warning and the clinical workload. ➀ Graded visual distinction mechanism: High-risk alarms (such as the use of metformin in combination with contrast agents, severe renal insufficiency) are presented with prominent red pop-up windows that cannot be skipped; medium-risk safety reminders use embedded prompts within the page, without imposing strict restrictions, and serve as early warning alerts; low-risk reminder information is displayed in the sidebar of the electronic medical record system for clinicians to view at their discretion. ➁ Simplified design of core information: All alarm contents only retain the key risk factors and corresponding one-click executable intervention suggestions, eliminating redundant descriptive text, and reducing the reading burden on clinicians. ➂ Integration and suppression mechanism for repeated alarms: For repeated medication instructions for the same patient within 24 h, the system will automatically merge them into a single valid reminder to avoid frequent interference. By tracking the alarm situations through the system’s background data, it was found that compared with the uniform and indiscriminate alarm alerts in the conventional information-based methods, the hierarchical risk-triggering mechanism in the digital intelligence closed-loop management can significantly reduce the number of triggered alarms. Moreover, based on the actual situation, it presents efficient alarm methods such as risk reminders, early warnings, and intelligent intercepts to assist doctors in clinical decision-making. Therefore, through the multi-layer alarm management strategy of “source filtering—hierarchical presentation—integration suppression,” while ensuring that key risks are not missed, it effectively reduces the number of non-operational alarms and lowers the cognitive load and interruption frequency in clinical decision-making. The background data tracking also confirmed that compared with the uniform and indiscriminate reminder mode of the conventional information-based methods, the hierarchical risk-triggering mechanism in the digital intelligence closed-loop management significantly reduces the frequency of invalid alarms. At the same time, through the layered collaboration of risk reminders, early warnings, and intelligent intercepts, clinical doctors can obtain appropriate information at the appropriate time, which not only improves decision-making efficiency but also strengthens the patient safety defense line. This result indicates that alarm management should not be limited to “whether there is an alert,” but should focus on “when, in what way, to whom, and to what extent to give an alert.” Only by embedding the alarm logic into clinical scenarios and continuously optimizing its presentation method can we truly solve the alarm fatigue problem and achieve the organic integration of technology and clinical workflow. Therefore, based on the clinical scenario-driven hierarchical risk triggering and interface optimization strategy, it can effectively alleviate alarm fatigue without sacrificing patient safety, and achieve the transformation from “information bombardment” to “precise warning.”

### 4.5 The scalability and adaptability of digital logic rules in different electronic health record architectures

We acknowledge that this study was conducted within a single healthcare institution, which may limit the direct generalizability of our research results in other healthcare environments. There are significant differences among hospitals and healthcare systems in terms of their workflow, the degree of integration of digital infrastructure, and the compliance rates of medical staff. However, we believe that the digital logic rules developed in this study have inherent characteristics that may promote their scalability and adaptability in heterogeneous electronic health record (EHR) environments. Firstly, the logical framework of our rules is based on the “if… then…” system logic, which links multiple systems together to form an automated rule engine with an “identification-decision-execution” process. The core is the rule reasoning engine of the clinical decision support system. These rules are not hardcoded in specific data fields or the proprietary database schema of the institution’s electronic medical record (EHR) system, but are built around the standard clinical concepts commonly available in most electronic medical record platforms (such as diagnostic codes, laboratory results, medication prescriptions, etc.). This modular design enables local data elements to be directly mapped to the rule input parameters, requiring only a small amount of site-specific configuration without the need for fundamental rule reconfiguration. Secondly, our rule engine adopts a layered architecture, decoupling clinical decision logic from the underlying data access layer. This design conforms to the best practices for the interoperability of clinical decision support systems and allows the same rule logic to be deployed on different EHR backends, only requiring the replacement of the data extraction module while retaining the core reasoning algorithm. However, we openly admit that successful adaptation to different institutional environments requires not only technical interoperability. Organizational factors—including local workflow patterns, personnel training needs, and the readiness of the institution for digital transformation—also play an important role in determining the actual implementation effectiveness. The significant differences in doctor compliance rates in different clinical practice environments highlight the need to develop implementation strategies tailored to local conditions rather than adopting a one-size-fits-all promotion approach.

### 4.6 Limitations

However, this study has certain limitations. For instance, as a single-center study, the generalizability of the conclusions needs to be carefully evaluated. This intervention measure helped us reduce the metformin medication errors after CTA examination by six times. However, at the same time, other factors that might have reduced medication errors during the study period were not taken into account. Firstly, this study adopted a single-group pre-post design, without setting up a matched parallel control group, which made the results susceptible to significant historical bias and unmeasured confounding variables. For example, during the study period, we did conduct regular medication safety training for medical staff and implement standardized nursing education, which might also be conducive to helping reduce the occurrence of medication errors. These are also factors that cannot be ignored. During the study period, the staffing structure and shifts of medical staff were always in a dynamic adjustment state. Although the overall personnel turnover rate was small, this was also a factor that needed to be taken into consideration. Additionally, the fundamental cause analysis of previous medication events, combined with the continuous promotion of routine medical quality supervision and dispensing process optimization by hospital management, also significantly contributed to enhancing the risk prevention awareness of medical staff. All these organizational factors are embedded in the real clinical workflow and should not be separated from the outcome of the digital and intelligent intervention effect. The limitations of this study also include that no sample size estimation process was pre-determined in the early stage of the research, and the target test power was not calculated in the research design stage. In the future, similar prospective studies can complete the calculation of sample size and confidence level in advance, and further standardize the statistical design. Therefore, in the subsequent follow-up studies, it is necessary to adopt a controlled trial with a matched control ward or a historical matched cohort to quantify the independent contribution of the digital and intelligent intervention measures. This study can only confirm that in the complex hospital social-technical ecosystem, the deployment of digital and intelligent intervention occurs simultaneously with a significant decrease in the medication error rate, and the digital and intelligent intervention method is used as an auxiliary technical means for medication safety management, rather than the sole decisive factor.

## 5 Conclusion

This study systematically and effectively reduced the risk of metformin medication errors within 48 hours after CTA examination through digitalization intervention methods, improving medication safety and management efficiency. This intervention also provides a reference for preventing other types of doctor’s orders execution errors or process-related adverse events. Although this study has verified the feasibility and initial efficacy of our digital logic rules in a single-center environment, we recognize that their broader clinical application requires rigorous validation in different medical settings. In the future, it is recommended to conduct a prospective multi-center implementation study to evaluate the portability, adaptability, and sustained effectiveness of this rule system in different EHR architectures, institutional workflows, and user groups. At the same time, we should further explore the application of information technology in risk assessment and early warning, promoting the scientific development of adverse event prevention and management, thereby ensuring patient safety and providing higher-level evidence-based evidence for future related research.

## Data Availability

The original contributions presented in the study are included in the article/supplementary material, further inquiries can be directed to the corresponding authors.
